# Epoxyscillirosidine Induced Cytotoxicity and Ultrastructural Changes in a Rat Embryonic Cardiomyocyte (H9c2) Cell Line

**DOI:** 10.3390/toxins11050284

**Published:** 2019-05-21

**Authors:** Hamza Ibrahim Isa, Gezina Catharina Helena Ferreira, Jan Ernst Crafford, Christoffel Jacobus Botha

**Affiliations:** 1 Department of Paraclinical Sciences, University of Pretoria, Onderstepoort 0110, Gauteng, South Africa; arina.ferreira@up.ac.za (G.C.H.F.); christo.botha@up.ac.za (C.J.B.); 2 Department of Veterinary Pharmacology and Toxicology, Ahmadu Bello University, Zaria 810107, Nigeria; 3 Department of Veterinary Tropical Diseases, University of Pretoria, Onderstepoort 0110, Gauteng, South Africa; jannie.crafford@up.ac.za

**Keywords:** Cardiac glycoside, epoxyscillirosidine, H9c2 cells, hormesis, LDH assay, *Moraea pallida*, MTT assay, necrosis, poisoning

## Abstract

*Moraea pallida* Bak. (yellow tulp) poisoning is the most important cardiac glycoside-induced intoxication in ruminants in South Africa. The toxic principle, 1α, 2α-epoxyscillirosidine, is a bufadienolide. To replace the use of sentient animals in toxicity testing, the aim of this study was to evaluate the cytotoxic effects of epoxyscillirosidine on rat embryonic cardiomyocytes (H9c2 cell line). This in vitro cell model can then be used in future toxin neutralization or toxico-therapy studies. Cell viability, evaluated with the methyl blue thiazol tetrazolium (MTT) assay, indicated a hormetic dose/concentration response, characterized by a biphasic low dose stimulation and high dose inhibition. Increased cell membrane permeability and leakage, as expected with necrotic cells, were demonstrated with the lactate dehydrogenase (LDH) assay. The LC_50_ was 382.68, 132.28 and 289.23 µM for 24, 48, and 72 h respectively. Numerous cytoplasmic vacuoles, karyolysis and damage to the cell membrane, indicative of necrosis, were observed at higher doses. Ultra-structural changes suggested that the cause of H9c2 cell death, subsequent to epoxyscillirosidine exposure, is necrosis, which is consistent with myocardial necrosis observed at necropsy. Based on the toxicity observed, and supported by ultra-structural findings, the H9c2 cell line could be a suitable in vitro model to evaluate epoxyscillirosidine neutralization or other therapeutic interventions in the future.

## 1. Introduction

*Moraea pallida* Bak. (yellow tulp) poisoning in livestock is the most important of all cardiac glycoside-associated plant poisonings in the Republic of South Africa. Acute poisoning with severe cardiac rhythm aberrations occurs, and the mortality rate is high. Yellow tulp poisoning together with other cardiac glycoside toxicoses accounts for about 33% and 10% mortality, due to plant poisonings in cattle, and small stock, respectively [1]. In poisoned animals the respiratory, cardiovascular, gastrointestinal, and nervous systems are involved, and signs may include general apathy, tremors, weakness of hindquarters, respiratory distress, and at times bruxism and groaning sounds [2]. Microscopic cardiac lesions are myocardial degeneration and necrosis [3]. The toxic principle is a bufadienolide, 1α, 2α-epoxyscillirosidine [3,4]. The molecular formula of the compound is C_26_H_32_O_8_ and is chemically closely related to scillirosidine, contained in *Urginea maritima* var *rubra*. Epoxyscillirosidine was identified as being responsible for the intoxication of livestock [4], as well as an aversive compound, causing feed aversion in livestock [3,5,6]. Bufadienolides, similar to other cardiac glycosides, interfere with the function of the ubiquitous sodium potassium adenosine triphosphatase (Na^+^-K^+^-ATPase) on the cell membrane [7]. The Na^+^-K^+^-ATPase acts as the receptor for cardiac glycosides and structurally similar compounds [7,8]. 

The H9c2 (2-1) embryonic rat cardiomyocyte cell line is a sub-clone of the original clonal line, which was derived from embryonic BD1X rat cardiac tissue [9]. This cell line has commonly been utilized in cardiotoxicity studies of novel, mainly anticancer drugs, to elucidate mechanisms of cell injury in cardiac cells, and to evaluate apoptotic and necrotic lesions in cardiomyocytes, that are induced by various compounds and toxins [10]. 

The response of the H9c2 cells, following epoxyscillirosidine exposure, may reveal important clues as to how cardiac cells and the heart are affected in animals poisoned by yellow tulp. In addition, an in vitro tissue culture model can replace the use of sentient animals and circumvent animal ethics concerns in future toxicity studies. The aim of this study was to investigate the effect of epoxyscillirosidine on rat embryonic cardiomyocytes of the H9c2 type by evaluating cell viability, cytotoxicity, as well as characterizing morphological changes induced in exposed cells. Cell viability was evaluated using the methyl blue thiazol tetrazolium (MTT) assay, while cytotoxicity was determined using the lactate dehydrogenase (LDH) release assay. Ultra-structural changes were assessed using transmission electron microscopy (TEM).

## 2. Results

### 2.1. Cell Viability

**Figure 1:**
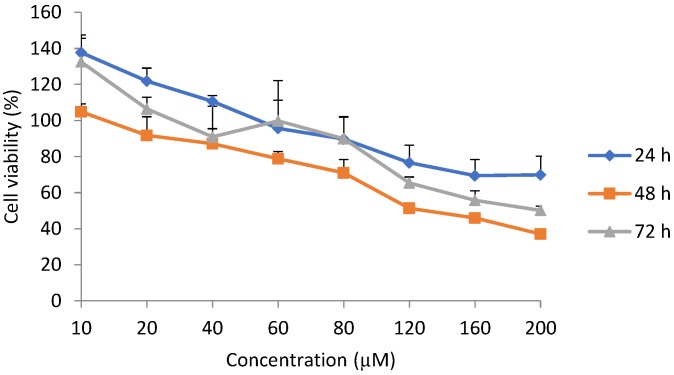
Dose-response curve for viability using methyl blue thiazol tetrazolium (MTT) assay in H9c2 cells exposed to epoxyscillirosidine for 24, 48 and 72 h. Cell viability presented as percentage of control (*n* = 3). Each value in the curves represents per-cent viability (mean ± SD). The Students; *t*-test (unpaired, two tailed) was used to compare the durations of exposure. Cell viability was not significantly (*p* > 0.05) different between any of the durations (24, 48 and 72 h). The values were the average of three independent experiments.

Cell viability increased when the H9c2 cells were exposed to epoxyscillirosidine at low doses (10–40, 10 and 10–20 µM) for 24, 48 and 72 h, respectively. After 24 h, the highest (relative to control) cell viability (137.68 ± 7.87%) was observed following exposure to 10 µM and the toxic effect started manifesting at 60 µM (95.62 ± 15.68% cell viability). The lowest cell viability (69.42 ± 8.95%) observed was at 160 µM (Figure 1). 

After 48 h exposure, at 10 µM, cell viability was 104.85 ± 4.32%. The toxic effects of epoxyscillirosidine were seen at 20 µM, with cell viability of 91.75 ± 10.35% (Figure 1). Cell viability was 37.04 ± 2.34% at the highest dose (200 µM) evaluated. 

After 72 h incubation at 10 µM epoxyscillirosidine the cell viability was 132.52 ± 14.89%. The toxic effect started manifesting only at 40 µM, where cell viability decreased to 90.90 ± 17.06%. At 200 µM, the cell viability was 50.16 ± 2.31% (Figure 1).

Overall, cell viability was not significantly (*p* > 0.05) different among the three durations.

### 2.2. Median Lethal Concentration

**Figure 2:**
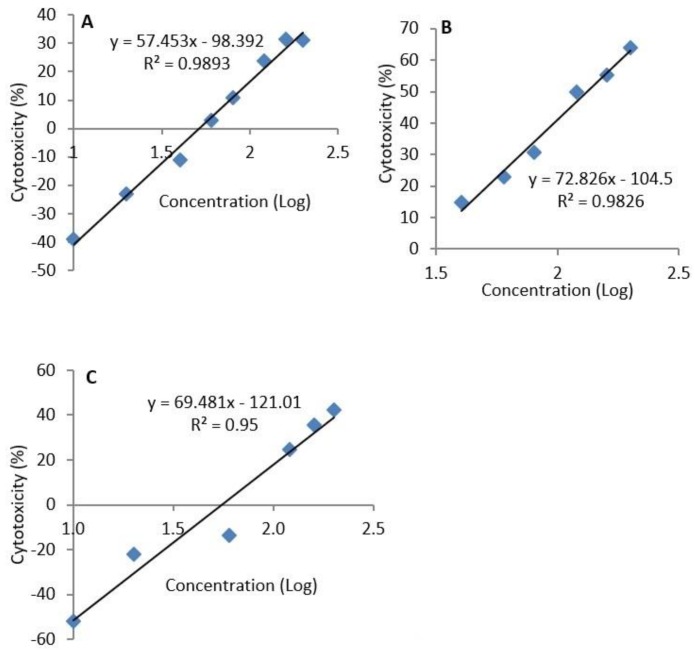
Semi-logarithmic concentration-cytotoxicity plots for H9c2 cells exposure to epoxyscillirosidine for 24, 48 and 72 h (**A**–**C**, respectively). The MTT assay was used to evaluate cytotoxicity. The LC_50_ was determined by substituting the value for *y* with 50 in the equation *y = mx + c*. The LC_50_ is the antilog of the value obtained for *x*. The result represents the means of 3 independent experiments.

The median lethal concentration (LC_50_) of epoxyscillirosidine, was extrapolated to be 382.68, 132.28 and 289.23 µM after 24, 48, and 72 h, respectively (Figure 2). 

### 2.3. Cytotoxicity

**Figure 3:**
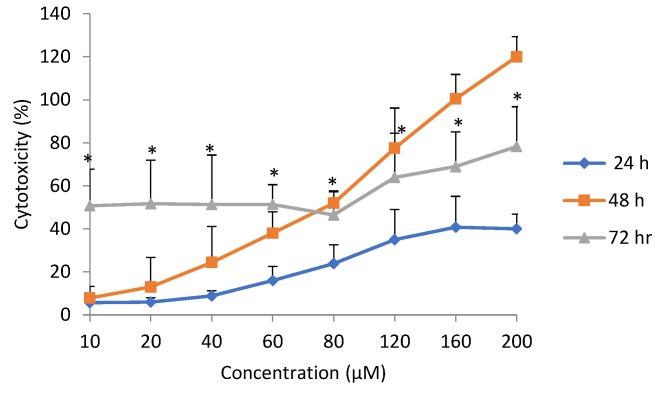
Dose-response curve for cytotoxicity (lactate dehydrogenase (LDH) assay) in H9c2 cells exposed to epoxyscillirosidine for 24, 48 and 72 h. Each value in the curve represents cytotoxicity as percentage (mean ± SD). Student’s *t*-test (unpaired, two tailed) was used to compare the durations of exposure. *Cytotoxicity at 72 h was significantly (*p* < 0.05) different from than at 24 h. The values are the result of three independent experiments.

The cytotoxicity following exposure to epoxyscillirosidine, as evaluated using the LDH release assay, revealed a dose- and time-dependent effect (Figure 3). Cytotoxicity (expressed as percentage of total lysed cells) increased from 5.69 ± 1.79% after 24 h to 50.73 ± 16.97% after 72 h at the lowest dose (10 µm), while it ranged from 40.02 ± 6.82 to 78.25 ± 18.52% at the highest dose (200 µM) evaluated.

Cytotoxicity at the same dose, but at different exposure durations, revealed a time-dependent effect. After 24 h exposure to epoxyscillirosidine, the lowest percentage cytotoxicity across all the different doses occurred (Figure 3). The 48 h duration showed lower percentage cytotoxicity when compared with 72 h at lower doses up to 60 µM, but the effect was higher from 80–200 µM. Cytotoxicity at 72 h was significantly (*p* < 0.05) different from that at 24 h.

**Figure 4:**
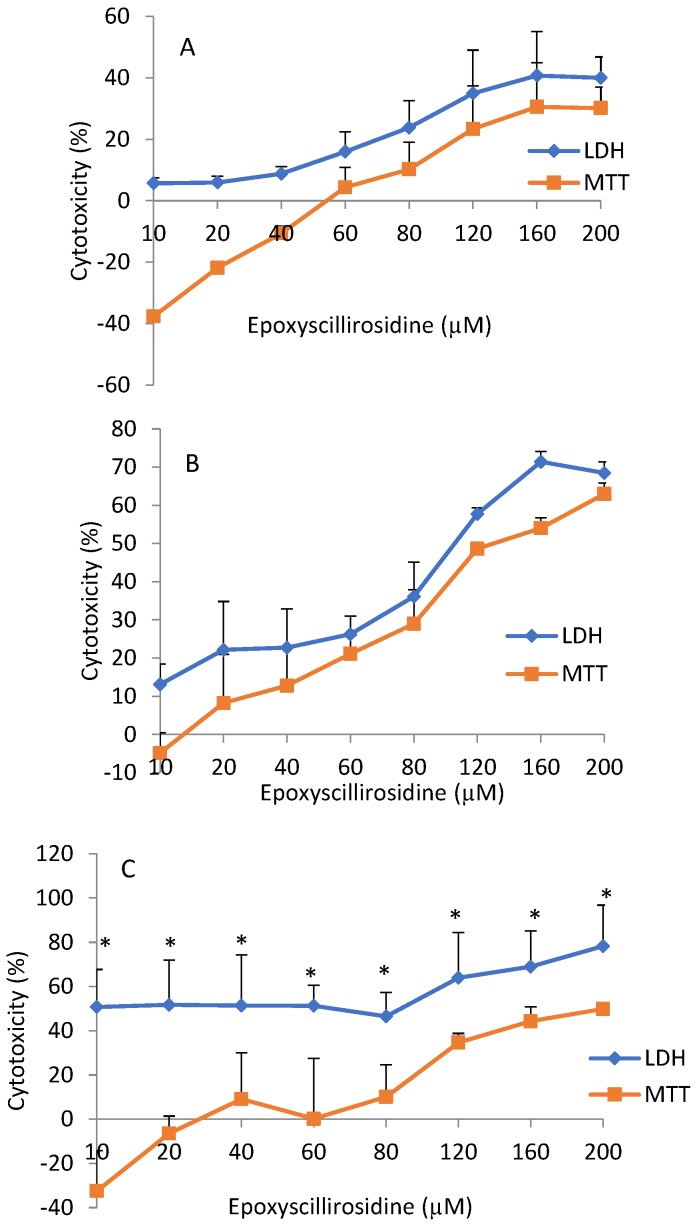
Comparison of the MTT and LDH dose-response curves of H9c2 cells exposure to epoxyscillirosidine for 24, 48, and 72 h (**A**–**C**, respectively). The negative MTT values represent a stimulatory effect and cell proliferation at low doses (hormesis). Each value in the curve represents percentage (mean ± SD) cell viability (relative to control). Student’s *t*-test was used to analyze the data. Cytotoxicity using LDH compared with MTT assay was significantly (*p < 0.05) different at 72 h. There was no difference (*p* > 0.05) in cytotoxicity, as revealed by LDH and MTT assays, at 24 and 48 h. The values are the means of three independent experiments.

Cytotoxicity, as revealed by the MTT assay, was not significantly (*p* > 0.05) different from the LDH assay at 24 and 48 h. However, there was significant (*p* < 0.05) difference in cytotoxicity revealed by the LDH assay when compared with the MTT assay at 72 h (Figure 4).

Comparison of cytotoxicity using MTT and LDH assays at the same concentration and duration of exposure indicated that the LDH assay had consistently higher values than the cytotoxicity revealed by the MTT assay. For example, following exposure to 80 µM epoxyscillirosidine for 24 h, cytotoxicity was 23.83 ± 8.78% and 10.27 ± 12.48% with LDH, and MTT assays, respectively (Figure 4). Cytotoxic effect as evaluated with the MTT assay was negative at low doses in all the durations (Figure 4).

### 2.4. Changes in Ultra-Structure

**Figure 5:**
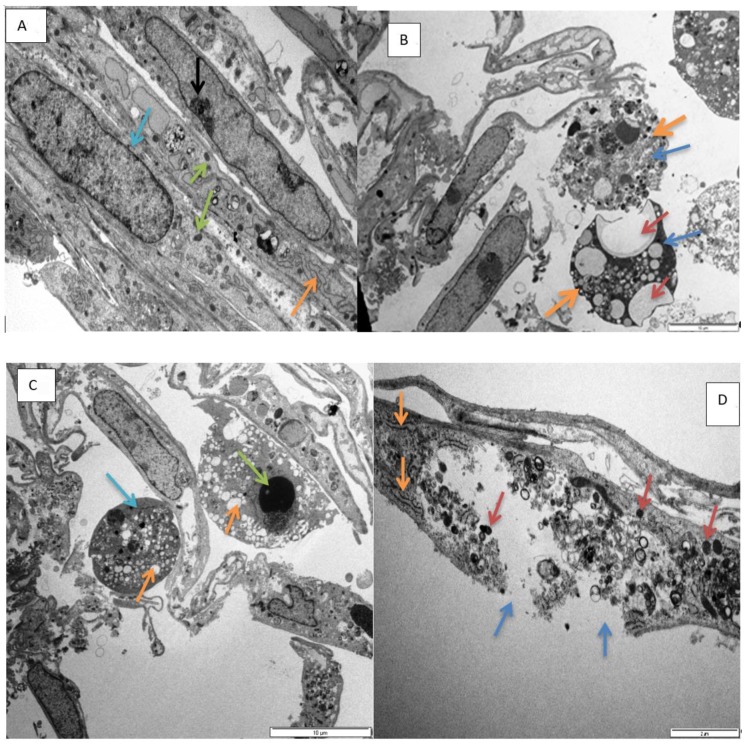

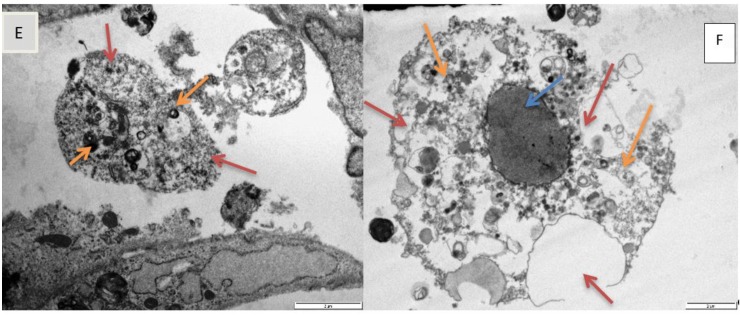
Transmission electron micrograph showing, (**A**). nucleus (blue arrow), mitochondria (green arrows), and endoplasmic reticulum (ER, orange arrow) in normal H9c2 cells (72 h control). Scale bar 5 µm. (**B**). Micrograph showing vacuolation (brown arrows), diffuse dissolution of the cytoplasm (blue arrows), spherical shaped cells (thick orange arrows) in H9c2 cells exposed to epoxyscillirosidine (160 µM) after 24 h. Scale bar 10 µm. (**C**). Micrograph showing karyolysis (green arrow), vacuolation (orange arrows) and diffuse dissolution of the cytoplasm and organelles (blue arrow) in H9c2 cells (200 µM, 24 h). Scale bar 10 µm. (**D**). Micrograph showing damaged plasma membrane (blue arrows), numerous vesicles containing electron dense material (brown arrow) and ER (orange arrows) in H9c2 cells (120 µM, 48 h). Scale bar 2µm. (**E**). Micrograph showing diffuse dissolution of the cytoplasm (blue arrow) and vesicles containing electron dense material (orange arrows) in amoeboid mis-shaped H9c2 cells (40 µM, 72 h). Scale bar 2 µm. (**F**). Micrograph showing karyolysis (blue arrow), vacuolation (brown arrows) and diffuse dissolution of the cytoplasm (orange arrows) in H9c2 cells (200 µM, after 72 h). Scale bar 2 µm.

The organelles and cell shape were unaffected in the control cells. Normally, H9c2 myoblasts are spindle-to-stellate-shaped cells that can be mono- or multi-nucleated [10] (Figure 5A). In cells exposed to epoxyscillirosidine over 24, 48, and 72 h, time and dose-dependent morphological alterations were observed. Appearance of cytoplasmic vesicles, vacuoles, changes in nuclear morphology, cytoplasm and damage to the cell membrane in H9c2 cells, after exposure to epoxyscillirosidine, were evident. These changes only became apparent at higher doses (160–200 µM) (Figure 5B,C,F). The cells also changed shape from spindle-like to spherical at higher concentrations (Figure 5B,C,E,F).

Furthermore, varying degree of karyolysis were observed in different exposures at higher doses (80–200 µM) (Figure 5C,F). Further nuclear alterations, induced by epoxyscillirosidine exposure, were also observed. Chromatin condensation, a typical marker of apoptosis, was also observable in some cells, most likely as a result of normal physiological function, due to senescence and ageing in H9c2 cells, which may not be related to epoxyscillirosidine exposure (not shown).

The cell membrane was interrupted, with wide spaces of discontinuity at higher concentrations (120–200 μM) at 48 (Figure 5D) and 72 h (not shown). Furthermore, increased cytosolic vacuoles were observed at these exposures (Figure 5F).

## 3. Discussion

This report describes the in vitro cytotoxic effect of epoxyscillirosidine on H9c2 cells. At 24, 48, and 72 h following exposure to epoxyscillirosidine, a dose and time dependent effect was observed. Interestingly, at low doses, epoxyscillirosidine stimulates cell viability. Weyermann et al., [11] reported increased values (above 100%) for viability, using MTT assay at low doses following exposure to sodium azide in mouse fibroblasts L (tk-) cells. This observation could be explained by hormesis, whereby a stress agent that is injurious to a biological system at high doses, produces a stimulatory effect at low doses [12,13]. Thus, the agent causes low dose stimulation and high dose inhibition of the observed/measured end point. Several in vitro studies have reported hormesis in different compounds, including the cardiac glycoside ouabain [14,15,16]. There are two major mechanisms that have been explained. It has been postulated that hormesis occurs as a compensatory mechanism, following exposure to any stress agent, and the mechanism is similar regardless of the level of organization (i.e, cell, tissue, organ, or whole organism), the nature of the stress agent (i.e, chemical, physical, or biological) or the endpoint measured (viability, cell proliferation, or death). The two pathways reported to mediate hormesis, are the signaling and the receptor mediated pathways [16]. It was reported that hormesis causes an increase to a factor less than two, and a maximal increase of 30–60% relative to control values [14]. The mechanism through which hormesis was mediated was, however, not investigated in this study. Ouabain, a cardenolide-type cardiac glycoside, was reported to induce hormesis through both receptor [17] and signaling pathway [18] systems.

The lowest viability was detected after 48 h. At 24 h, the time is probably too shorter to elicit a high toxic effect, resulting in higher viability relative to 48 h. Pathological effects following exposure of cells to chemicals/stressors have been reported to be dose and time dependent in in vitro studies [19,20,21,22]. However, at 48 h, more cells were damaged and hence the relatively lower viability. At 72 h, the unaffected cells have likely proliferated, following 96 h of seeding. This may explain the reason why the viability at 72 h was higher than that at 48 h.

The median lethal concentrations differed with exposure duration. At 72 h, the LC_50_ was higher than at 48 h, but lower than at 24 h. This could be explained by the fact that, at 72 h, the cells that were not affected by the toxin had proliferated and increased in number. Normally, H9c2 cells reach about 70–80% confluence 72–96 h following seeding. The values of LC_50_ have been reported to be influenced by exposure times according to the different experimental settings [23]. In contrast, in another study [24], the EC_50_ values reported for epoxyscillirosidine were much lower (41.39 ± 4.37, 25.42 ± 3.73 and 12.65 ± 2.75 µM for 24, 48 and 72 h exposures, respectively). It should be noted that LC_50_ just like LD_50_ is not a biologic constant. In fact, LC_50_ values could be influenced by different factors depending on the experimental conditions [23].

The cytotoxicity, using the MTT and LDH assay, did not yield similar results. The LDH assay showed a higher degree of variability, while the MTT assay produced more consistent values. Bopp and Lettieri [25] had similarly reported higher intra- and inter-assay variabilities for LDH, compared to the MTT assay and two other fluorometric methods. Cytotoxicity as measured with the LDH assay produced higher values even at low doses (Figure 3), whereas low doses stimulated cell viability as determined by the MTT assay. This may be explained by the fact that the LDH assay measured all dead cells, including those that may have died physiologically. In addition, newly damaged cells retain residual mitochondrial dehydrogenase activity, which could contribute to the metabolism of MTT in non-viable cells [26]. On the other hand, cytotoxicity is not exactly the opposite of cell viability. A cell may be exposed to an injurious agent, without the cell dying, but only experiencing some compromise in cellular functions. Thus the term ‘cell vitality’ refers to the physiological capabilities of cells [27], a different phenomenon from viability. The MTT assay determines viability, which is a positive phenomenon in metabolically active cells [28]. Furthermore, at low doses, cell viability was stimulated (hormesis), and thus, the lowered cytotoxicity at lower doses, as captured by the MTT assay, was possibly due to metabolic activation, resulting in higher than expected values for viability. It was hypothesized that metabolic activation was responsible for the increased viability, due to chitosan/sulphated locust bean nano-particle exposure in Caco-2 cells, as revealed by MTT assay [29].

Electron microscopy has provided insight into the morphological alterations, caused by exposure to epoxyscillirosidine for 24, 48, and 72 h. At the lowest dose evaluated (40 μM), no noteworthy ultrastructural alterations were observed. This is supported by the results of the MTT assay, where decreased viability only started manifesting from 60 µM epoxyscillirosidine (at 24 h). However, the highest dose (200 μM) was characterized by numerous cytoplasmic vacuoles and significant nuclear alterations, which include chromatin dissolution (karyolysis, Figure 5C,F) and disruption of the nuclear envelope (Figure 5D). These features are typical of necrosis. The observed morphological changes at higher doses (160–200 µM) are supported by the LDH assay findings, where LDH leakage through the damaged cell membranes, was demonstrated. Since the toxin interferes with and disrupts the sodium potassium ATPase on membrane of cells [7,8], it causes disturbance in ionic homeostasis and subsequently necrosis. In corroboration of this study, Henn et al. [24] reported mis-shaped nuclei and damage to the cell membrane in cells exposed to epoxyscillirosidine. The finding is consistent with myocardial necrosis observed microscopically in poisoned livestock [3].

In summary, following exposure of H9c2 cells to different concentrations of epoxyscillirosidine, the dose-response relationship indicated a hormetic effect, where low doses stimulated cell viability. The median lethal concentration of epoxyscillirosidine in rat embryonic cardiomyocytes (H9c2) as calculated, varied with 24 h being the highest, followed by 72 and 48 h in that order. The LC_50_ was 382.68, 132.28, and 289.23 µM for 24, 48, and 72 h, respectively. Ultra-structural changes showed that the cause of H9c2 cell death, subsequent to epoxyscillirosidine exposure, is necrosis. Increased cell membrane permeability and leakage of content, as expected with necrotic cells, was confirmed with the LDH release assay. Based on the findings from this study, the H9c2 cell line is a suitable in vitro model to study the effect of epoxyscillirosidine and could be used to study similar compounds.

## 4. Materials and Methods

### 4.1. Chemicals and Reagents

Purified epoxyscillirosidine (isolated according to the method of Naudé and Potgieter [30]) was available in the plant toxin collection of the Department of Paraclinical Sciences, Faculty of Veterinary Science, University of Pretoria, and was stored in a dried form in a refrigerator at 4 °C. Dimethyl sulphoxide (DMSO, cat. no: SAAR1865000LP) and Triton X-100 were obtained from Merck (Darmstadt, Germany). Trypsin-EDTA (cat no: BE17-16IF) and L-glutamine (cat no: BE-17-605E) were acquired from Lonza (Verviers, Belgium). The LDH assay kit (CytoTox-ONE^TM^, cat. no: G7890) was purchased from Promega Corp., USA. Dulbecco’s Modified Eagle’s Medium (DMEM, cat. no: D6546), phosphate buffered saline (PBS, cat. no: P4417), penicillin-streptomycin (cat. no: P4333), MTT (thiazolyl blue tetrazolium bromide) reagent (cat. no: M5655), and trypan blue (cat. no: T6146) were from Sigma-Aldrich (Darmstadt, Germany). Foetal bovine serum (cat. no: 10499-044, Gibco) was from Life Technologies (Grand Island, NY, USA).

### 4.2. Cell Culture

Rat embryonic cardiomyocytes (H9c2 (2-1) cells) [9] were purchased from the American Type Culture Collection (ATCC) (Manassas, Virginia, USA, cat no: CRL-1446^TM^). The cells were cultured in DMEM supplemented with 10% foetal bovine serum, 4 mM L glutamine and penicillin-streptomycin (100 U/mL) in 75 cm^2^ tissue culture flasks. Cells were maintained in a humidified incubator (HeraCELL 150^R^, Thermo-Electron Corporation, Waltham, MA, USA) in a 95% air 5% CO_2_ environment at 37 °C. Medium was changed every 3–4 days, while the cells were sub-cultured after attaining about 70–80% confluency. Cells were detached using trypsin-EDTA. Cells were seeded for cytotoxicity and ultrastructural studies in 96-well microtitre plates, and 12-well plates, respectively.

### 4.3. Cytotoxicity Studies

The cells were detached from the cultivation flasks, using trypsin-EDTA, and counted with a hemocytometer with the aid of trypan blue exclusion to determine viability. Three 96-well plates were seeded with 1 × 10^4^ cells per well (final volume 200 µL per well). A triplicate of wells in each plate contained only medium, with no cells which served as blank. The plates were incubated for 24 hours to allow for cell attachment and recovery. Thereafter, 10, 20, 40, 60, 80, 120, 160, and 200 µM concentrations of epoxyscillirosidine were added to the wells in triplicate. Cells cultured in growth medium only, representing 100% viability were used as negative control in all experiments. In the LDH release assay, Triton X-100 was added to separate cells in triplicate as positive controls. The three plates were incubated for 24, 48, and 72 h, respectively, before the evaluation of cell viability and cytotoxicity using MTT, and LDH assays, respectively. The assays were validated and optimized before adoption in each case. The doses selected were identified following a preliminary dose finding study. The experiments were repeated three times, at weekly intervals.

#### 4.3.1. Evaluation of Cell Viability

Cell viability, following exposure of H9c2 cells to varying concentrations of epoxyscillirosidine, was determined using the MTT assay [28]. This is a quantitative colorimetric assay, that spectrophotometrically measures the amount of purple formazan crystals, formed by dehydrogenases in living viable cells from the yellow tetrazolium salt MTT. In brief, the three plates were treated after 24, 48, and 72 h, respectively, as follows: 100 µL of the medium was transferred to the LDH assay plate and the remaining medium decanted and cells washed with PBS (200 µL per well), followed by the addition of fresh DMEM (200 µL per well). Thereafter, 20 µL MTT reagent was added serially into all wells. The plates were incubated for 2 h. The medium, containing MTT reagent, was decanted, followed by the addition of 100 µL DMSO and shaking on a microplate shaker for 5 min to dissolve formed formazan. The absorbance was read in a multi-reader (Synergy HT, BioTek^R^ EL808, Winooski, VT, USA) using the Gen 5 protocol at 570 nm versus 630 nm. Percentage cell survival was calculated using the formula:

Cell viability (%) = (Absorbance of epoxyscillirosidine/Absorbance of cells only) × 100

The median lethal concentration (LC_50_) of epoxyscillirosidine was calculated using the straight line equation of the log dose-response curve of cytotoxicity against concentration.

#### 4.3.2. Cytotoxicity Assay Using LDH Release

Cytotoxicity in H9c2 cells exposed to epoxyscillirosidine was evaluated using the LDH assay, with the aid of a commercial kit CytoTox-ONE™. This is a fluorometric method that estimates the number of dead cells present in multi-well plates. The CytoTox-ONE™ assay rapidly measures the release of LDH from cells with a damaged membrane. LDH released into the culture medium is measured with a 10 min coupled enzymatic assay, that results in the conversion of resazurin into a fluorescent resorufin product. The amount of fluorescence produced is proportional to the number of lysed cells. The assay was carried out according to the manufacturer’s instructions. Aliquots (100 µL) of medium, from all wells in the plate seeded with H9c2 cells, were transferred into a black Nunc 96 well plate. Reconstituted LDH substrate mixture (100 µL each) was added to the wells in the black plate, which was wrapped and shaken for 10 min. Stop solution was added and fluorescence was read at an excitation wavelength of 560 nm and an emission wavelength of 590 nm and at a fluorescence sensitivity setting of 40, in a Synergy HT, BioTek^R^ EL808 (Winooski, VT, USA) multi-reader. Per-cent cytotoxicity was calculated using the formula:

Cytotoxicity (%) = (experimental − blank)/(Triton X − blank) × 100

### 4.4. Evaluation of Ultrastructural Changes Using TEM

Three 12-well plates, in which coverslips were placed in each well, were seeded with 1 × 10^5^ cells per well (final volume 2 mL per well). The plates were incubated for 24 h for the cells to recover and stabilize. Thereafter, 40, 80, 120, 160, and 200 µM concentrations of epoxyscillirosidine were added to the wells in duplicate. Untreated cells were used as control. The three plates were incubated for 24, 48, and 72 h, respectively, before the cells were further processed for TEM.

The culture medium was carefully removed from the wells and replaced with 2 mL of 2.5% glutaraldehyde in 0.075 M sodium phosphate (NaPO_4_) buffer (pH 7.4) and left for 1 h. The coverslips were removed and placed in a separate holder. The cells were scraped off from the culture coverslips, transferred to 2 mL Eppendorf tubes, and centrifuged at 2356 *g* to form pellets. These, as well as the coverslips, were then rinsed 3 times in 0.075 M phosphate buffer for 10 min, and post-fixed for 1 h with 1% osmium tetroxide (OsO_4)_. Samples were then rinsed again in 0.075 M phosphate buffer for 10 min and then dehydrated serially in 30, 50, 70, 90% and three times with 100% ethanol. The samples were embedded in TAAB 812 epoxy resin [31], followed by ultra-microtome sectioning. The sections were contrasted with a 2% aqueous solution of uranyl acetate for 10 min and lead citrate [32] for 2 min and examined with a Philips CM10 (*Philips* Electron Optics, Eindhoven, The Netherlands) TEM

### 4.5. Data Analysis

Data were analysed using GraphPad Prism 7 for windows (version 7.03, GraphPad Prism Software Inc., La Jolla, CA, USA). Values were expressed as percentage of untreated control cells. Student’s *t*-test or ANOVA was used to evaluate statistical difference for paired, or multiple comparison between groups respectively. Statistical significance was set at *p* < 0.05.
